# Cellular hyper-excitability caused by mutations that alter the activation process of voltage-gated sodium channels

**DOI:** 10.3389/fphys.2015.00045

**Published:** 2015-02-17

**Authors:** Mohamed-Yassine Amarouch, Hugues Abriel

**Affiliations:** ^1^Materials, Natural Substances, Environment and Modeling Laboratory, Multidisciplinary Faculty of Taza, University of Sidi Mohamed Ben Abdellah-FesTaza, Morocco; ^2^Department of Clinical Research, University of BernBern, Switzerland

**Keywords:** Na_v_1.5-I141V, hyper-excitability, dilated cardiomyopathy, myotonia, erythromelalgia

## Abstract

Voltage-gated sodium channels (Na_v_) are widely expressed as macro-molecular complexes in both excitable and non-excitable tissues. In excitable tissues, the upstroke of the action potential is the result of the passage of a large and rapid influx of sodium ions through these channels. Na_V_ dysfunction has been associated with an increasingly wide range of neurological, muscular and cardiac disorders. The purpose of this review is to summarize the recently identified sodium channel mutations that are linked to hyper-excitability phenotypes and associated with the alteration of the activation process of voltage gated sodium channels. Indeed, several clinical manifestations that demonstrate an alteration of tissue excitability were recently shown to be strongly associated with the presence of mutations that affect the activation process of the Na_v_. These emerging genotype-phenotype correlations have expanded the clinical spectrum of sodium channelopathies to include disorders which feature a hyper-excitability phenotype that may or may not be associated with a cardiomyopathy. The p.I141V mutation in *SCN4A* and *SCN5A*, as well as its homologous p.I136V mutation in *SCN9A*, are interesting examples of mutations that have been linked to inherited hyperexcitability myotonia, exercise-induced polymorphic ventricular arrhythmias and erythromelalgia, respectively. Regardless of which sodium channel isoform is investigated, the substitution of the isoleucine to valine in the locus 141 induces similar modifications in the biophysical properties of the Na_v_ by shifting the voltage-dependence of steady state activation toward more negative potentials.

## Introduction

**Figure 1:**
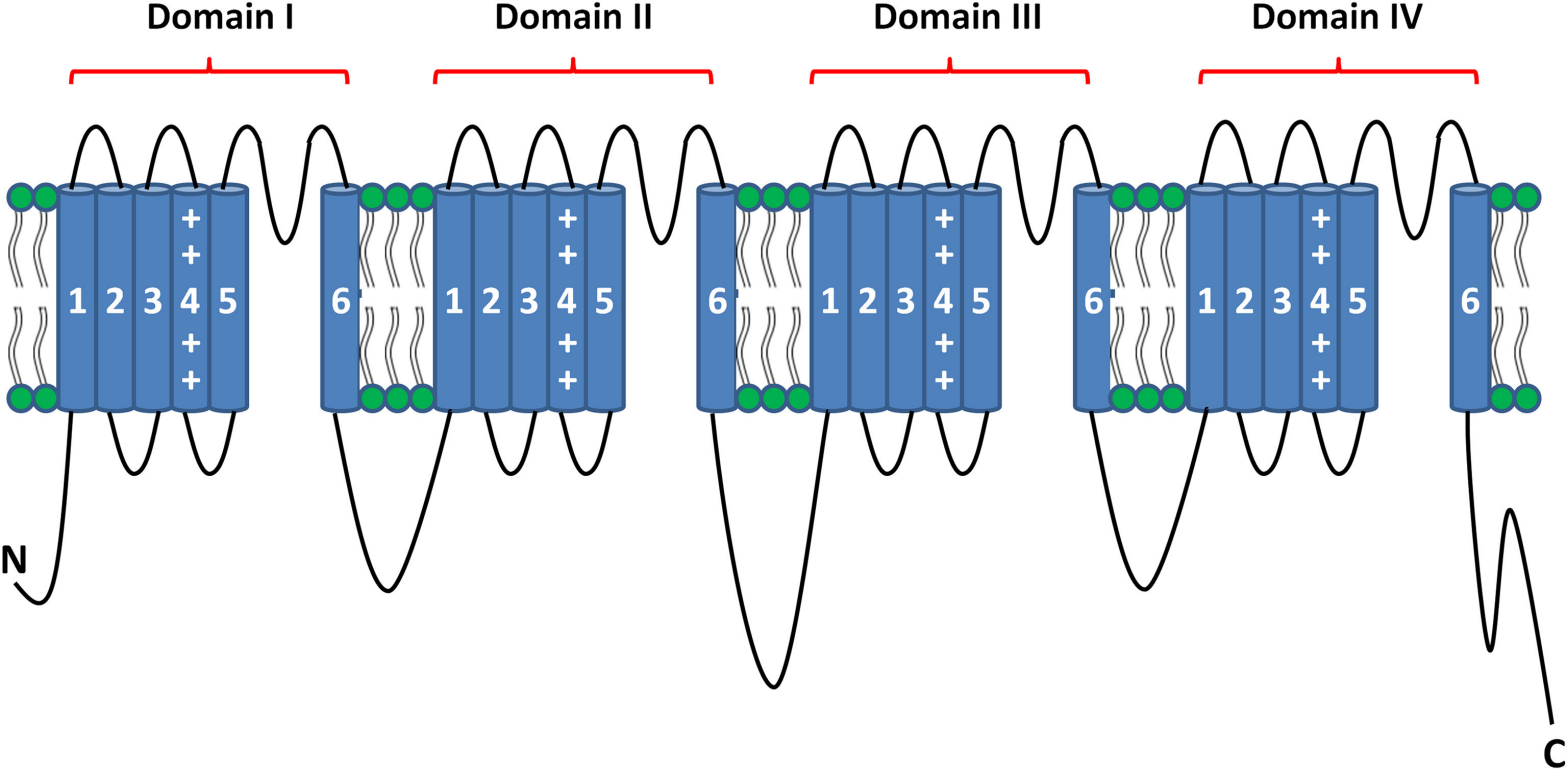
**Topology of the pore-forming α-subunit of voltage gated sodium channels**.

In excitable tissues, action potential initiation and propagation are the result of the passage of a large and rapid influx of sodium ions through the voltage-gated sodium channels (Na_V_). These channels consist of highly processed α-subunits that are present as nine different isoforms (Goldin et al., 2000). The α-subunit of the sodium channel is composed of four homologous domains (Noda et al., 1984). Each of these domains contains six α-helical transmembrane segments (S1–S6). The first four segments (S1–S4) comprise the voltage-sensing domain (VSD), and the last two segments (S5 and S6) form the pore of the channel when assembled in a tetrameric configuration (Figure 1) (Payandeh et al., 2011). Na_V_ dysfunction causes multiple inherited diseases, formerly known as channelopathies. Rare mutations and common variants in genes encoding the α-subunits have been associated with several familial forms of neurological, muscular and cardiac disorders (Cheng et al., 2008; Petitprez et al., 2008; Probst et al., 2009; Meisler et al., 2010; Abriel and Zaklyazminskaya, 2013; Bezzina et al., 2013; Liu et al., 2014; Swan et al., 2014).

The Na_V_ have been shown to be part of multi-protein complexes that are located in different cellular compartments. In addition to the Na_v_ α-subunits, these complexes have Na_v_-interacting proteins that regulate channel expression and function (Abriel, 2010; Laedermann et al., 2013a,b). Similar to that described for the sodium channel α-subunits, mutation in genes encoding the Na_v_-interacting proteins have been linked to the occurrence of several inherited diseases (Abriel, 2010; Catterall, 2014).

Several naturally occurring mutations that affect the activation process of the voltage-gated sodium channel have been recently associated with alterations of neuronal, muscular and cardiac excitabilities (Cheng et al., 2008; Petitprez et al., 2008; Laurent et al., 2012; Mann et al., 2012; Beckermann et al., 2014). The p.I141V mutation in *SCN4A* and *SCN5A*, as well as its homologous mutation p.I136V in *SCN9A*, are interesting examples of substitutions that lead to the occurrence of inherited hyper-excitability phenotypes. Depending on the sodium channel isoform, the I/V substitution is associated with familial forms of myotonia, exercise-induced polymorphic ventricular arrhythmias or erythromelalgia (Lee et al., 2007; Cheng et al., 2008; Petitprez et al., 2008; Swan et al., 2014).

The purpose of this review is to summarize the recently identified sodium channel mutations that are linked to cardiac hyper-excitability phenotypes and associated with the alteration of the activation process of voltage gated sodium channels.

## Cellular excitability and voltage gated sodium channels

In nervous, muscular, and cardiac tissues, I_*Na*_ influx through the Na_v_ channels is the major depolarizing current and, thus, underlies cellular excitability. Mutations that affect the function of these Na_v_ channels have been shown to modify the excitability pattern of these tissues. As an example, loss of function mutations of Na_v_1.5, leading to a decreased excitability of cardiac cells (hypo-excitability), slow the cardiac conduction velocity (Amin et al., 2010). On the opposite, gain of function mutations of these channels may reduce the excitation threshold and increase the conduction velocity leading to an increased cardiac excitability (hyper-excitability) (Swan et al., 2014). In the present review article, we focus on mutations that are linked with cellular hyper-excitability phenotypes.

## Cardiac hyper-excitability phenotypes related to an altered activation process of Na_v_1.5

### The cardiac sodium channel, Na_v_1.5

Na_v_1.5 is the main sodium channel isoform expressed in cardiac cells (Yu and Catterall, 2003). Other Na_v_ α-subunits, such as Na_v_1.1, 1.3, 1.6, and 1.8, are also present in the heart (Maier et al., 2004; Yang et al., 2012), and are mainly localized on the cardiomyocytes T-tubules or in the intracardiac neurons involved in neural control of the heart (Maier et al., 2002, 2004). These “non-cardiac” channels contribute to the conduction of a small proportion of the cardiac sodium current (Maier et al., 2002, 2004).

As aforementioned, the cardiac sodium channel is a multiprotein complex in which auxiliary proteins (i.e., β subunits) interact with the α-subunit, Na_v_1.5, to regulate its biology and function (Abriel, 2010). Some of these proteins, which are localized in specific regions of cardiac cells, have been shown to interact with the same regulatory domain of Na_v_1.5 (Abriel, 2010; Shy et al., 2013). As demonstrated by Shy and colleagues (Shy et al., 2014), the Na_v_1.5 channels are expressed as at least two distinct functional pools that are localized at the intercalated discs and the lateral membranes of the cardiomyocyte (Shy et al., 2014).

### Dilated cardiomyopathy and ion channel dysfunction

Dilated cardiomyopathy (DCM) is a cardiac structural disease characterized by decreased systolic function and ventricular dilatation. Inherited forms of this structural abnormality have been mainly linked to mutations in genes coding for cytoskeletal proteins (Haas et al., 2014). DCM has also been associated with mutations that affect Na_v_1.5 function, providing support to the argument that DCM could be considered as one of the phenotypes of cardiac sodium channelopathy (McNair et al., 2004; Olson et al., 2005; Ge et al., 2008; Nguyen et al., 2008; Morales et al., 2010; Laurent et al., 2012; Mann et al., 2012; Beckermann et al., 2014; Haas et al., 2014). The identified mutations appear to be preferentially localized in the VSD region of Na_v_1.5, and induce a loss or gain of function by affecting the voltage-dependencies of steady state activation and/or inactivation (McNair et al., 2004; Ge et al., 2008; Nguyen et al., 2008; Laurent et al., 2012; Mann et al., 2012; Beckermann et al., 2014). The sodium currents generated by some of these mutants have larger sodium window current peaks that are shifted toward more negative potentials (Nguyen et al., 2008; Laurent et al., 2012; Mann et al., 2012; Beckermann et al., 2014). In addition, Gosselin-Badaroudine and colleagues (Gosselin-Badaroudine et al., 2012) demonstrated that the R219H mutation in Na_v_1.5 causes a proton leak current, suggesting that this mutation induces intracellular acidification which may contribute to the DCM phenotype (Gosselin-Badaroudine et al., 2012).

The majority of studies that link the *SCN5A* gene to the occurrence of DCM demonstrate that this phenotype is usually associated with alterations in cardiac excitability (McNair et al., 2004; Ge et al., 2008; Nguyen et al., 2008; Gosselin-Badaroudine et al., 2012; Laurent et al., 2012; Mann et al., 2012; Shen et al., 2013; Beckermann et al., 2014). This observation raises several questions about the real origin of the observed structural defects. Are they a direct consequence of alterations in Na_v_1.5 function, or a result of pre-existing electrical arrhythmias? In some studies, the results of pharmacological therapy support the second hypothesis. Laurent et al (Laurent et al., 2012) and Mann et al (Mann et al., 2012) demonstrated improvement in cardiac function using the sodium channel-blocking properties of some anti-arrhythmic drugs, such as amiodarone, flecainide, and quinidine (Laurent et al., 2012; Mann et al., 2012).

These observations suggest that the association between Na_v_1.5 mutations and DCM is multifactorial. Some of the known involved factors are the existence of long-standing arrhythmias, the alteration of sodium channel function, the genetic background of the patient, and the presence of structural abnormalities (McNair et al., 2004; Ge et al., 2008; Nguyen et al., 2008; Cheng et al., 2010; Gosselin-Badaroudine et al., 2012; Laurent et al., 2012; Mann et al., 2012; Shen et al., 2013; Beckermann et al., 2014).

### Cardiac hyper-excitability phenotypes associated with Na_v_1.5 voltage sensor mutations

**Figure 2:**
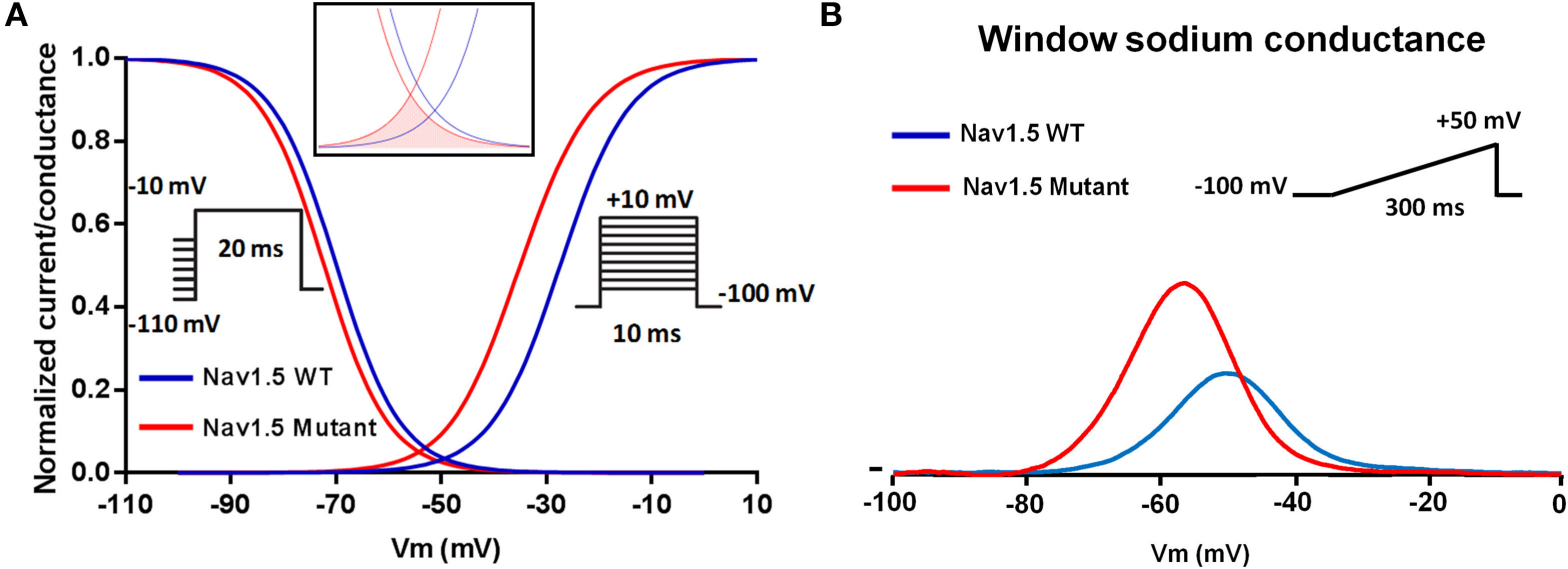
**Schematic representation of the shared mechanism of Na_v_1.5 mutations associated with cardiac hyper-excitability**. Negative shift of the voltage dependence of activation **(A)** leading to a negative shift of the sodium window conductance **(B)**.

Several studies (Olson et al., 2005; Laurent et al., 2012; Mann et al., 2012; Nair et al., 2012; Beckermann et al., 2014) have recently reported a new *SCN5A*-dependent clinical presentation characterized by an alteration in tissue excitability associated with DCM. All of the related *SCN5A* mutations (p.R814W, p.R222Q, p.R219H, and p.R225P) neutralize arginine residues that are localized in the S4 segment of domain I and II (Olson et al., 2005; Laurent et al., 2012; Mann et al., 2012; Nair et al., 2012; Beckermann et al., 2014). The functional consequences of these substitutions is either the alteration of Na_v_1.5 gating (Figure 2) (Olson et al., 2005; Laurent et al., 2012; Mann et al., 2012; Nair et al., 2012; Beckermann et al., 2014) or the induction of a pH-dependent inward H^+^ current (Gosselin-Badaroudine et al., 2012).

The p.R814W substitution was the first mutation linked to the neutralization of an arginine in the S4 segment of Na_v_1.5. This mutation was associated with the occurrence of cardiac hyper-excitability and DCM (Olson et al., 2005). When compared to the WT condition, the Na_v_1.5-R814W mutant negatively shifted the voltage dependence of activation, slowed activation kinetics and increased the sodium window current (Nguyen et al., 2008).

Similar biophysical modifications of Na_v_1.5 were observed for the p.R222Q mutation. This mutation shifted the voltage dependence of activation toward more negative potentials and hastened the activation kinetics. The voltage dependence of inactivation, when combined with the activation shift, increased and shifted the sodium window current toward more negative potentials (Laurent et al., 2012; Mann et al., 2012; Nair et al., 2012). The clinical phenotypes associated with the p.R222Q substitution were variable. The observed phenotypes included the occurrence of peripartum DCM, arrhythmic DCM, escape capture bigeminy, and multifocal ectopic Purkinje-related premature contractions associated with DCM (Olson et al., 2005; Morales et al., 2010; Laurent et al., 2012; Mann et al., 2012; Nair et al., 2012).

An original mechanism linking the neutralization of S4 arginine residues with the occurrence of cardiac hyper-excitability has been described for the p.R219H mutation (Gosselin-Badaroudine et al., 2012). The functional characterization of this mutation by Chahine et al suggested that the presence of the p.R219H mutation may induce intracellular acidification by creating a pH-dependent inward proton current, thus favoring the development of DCM and cardiac arrhythmias (Gosselin-Badaroudine et al., 2012).

Another Na_v_1.5 VSD arginine mutation, p.R225P, was recently identified in a boy with a prenatal arrhythmia and impaired cardiac contractility, followed by postnatal multifocal ventricular ectopy (Beckermann et al., 2014). This mutation affects the activation and inactivation processes, resulting in an increased and hyperpolarized sodium window current. The authors suggested that these biophysical modifications may lead to an aberrant sodium influx at potential ranges that are close to the resting membrane potential of cardiac cells, and thus may modify the excitability of cardiomyocytes (Beckermann et al., 2014).

### Exercise-induced polymorphic ventricular arrhythmias

A clinical and genetic study of a large multigenerational Finnish family recently demonstrated an inherited form of exercise-induced polymorphic ventricular arrhythmia caused by a newly identified *SCN5A* mutation, p.I141V (Swan et al., 2014). This mutation is located in a highly conserved region of the Na_v_1.5 channel domain I S1 transmembrane segment. The p.I141V mutation shifted the voltage dependence of steady state activation toward more negative potentials. The p.I141V window current exhibited a larger peak which was shifted toward more negative potentials as compared to the WT (Figure 2). Computer modeling of the biophysical modifications induced by the p.I141V mutation, however, suggested a reduced excitation threshold for action potential generation in the presence of this mutation as compared to the WT.

**Figure 3:**
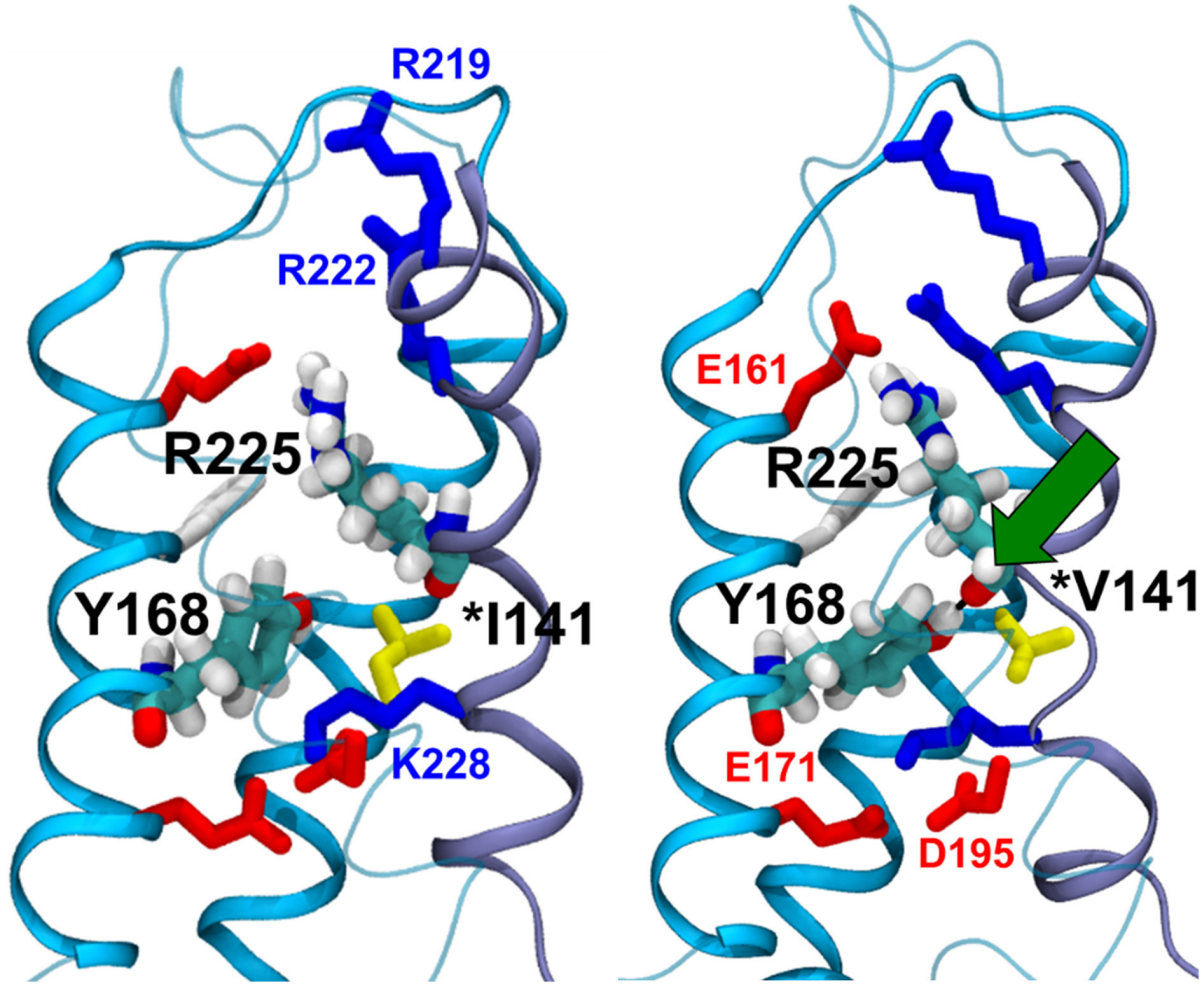
**Molecular dynamics simulation of the WT (left panel) and the p.I141V mutants (right panel) of Na_v_1.4 channel**. In the presence of the p.I141V mutation, the model predicted the formation of a hydrogen bond (Green arrow) between the S2-Y168 and S4-R225 residues, thus stabilizing the open confirmation of the channel; (From Amarouch et al., 2014).

**Figure 4:**
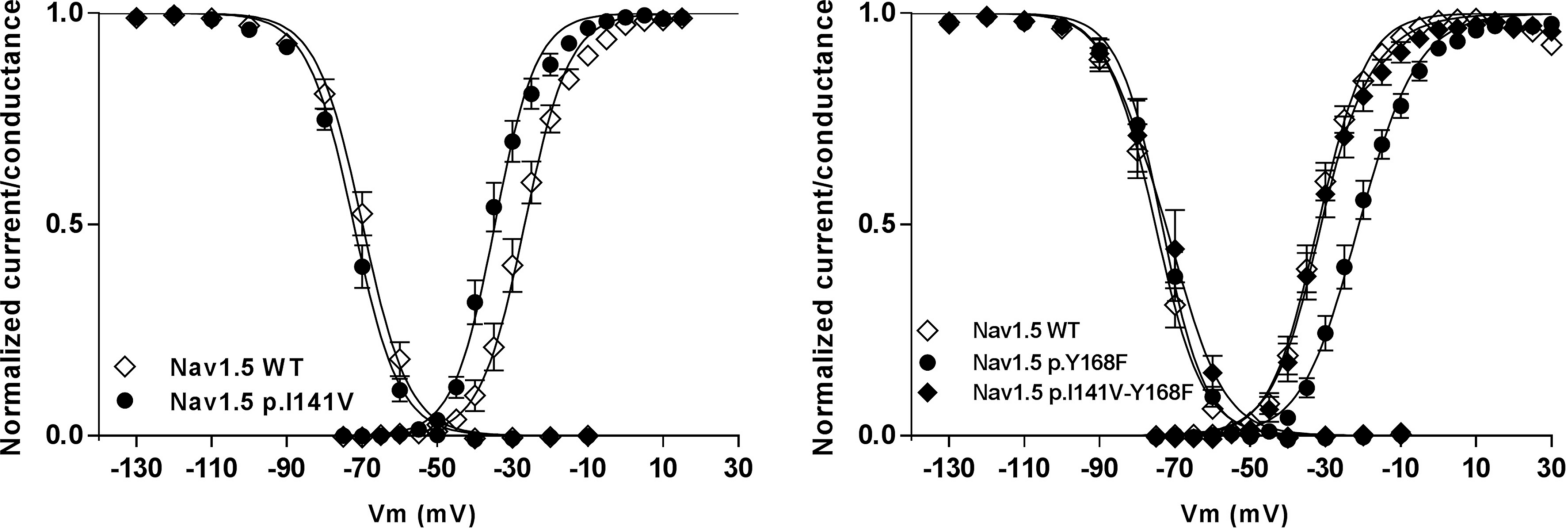
**The functional effect of the p.I141V mutation on the steady-state of activation and inactivation processes of Na_v_1.5 channel (left panel)**. The presence of this mutation induces a negative shift of the voltage dependence of activation. However, this effect was abolished in the presence of the p.Y168F substitution (right panel); (From Amarouch et al., 2014).

The crystal structure of the bacterial channel Na_v_Ab, published by the Catterall's group, shows close proximity between the isoleucine 141 residue of the S1 segment and arginines that are located in the S4 segment (Payandeh et al., 2011). Based on these observations, we hypothesized that the p.I141V substitution stabilizes the open conformation of the Na_V_ by modifying or creating new interactions between these specific segments (Amarouch et al., 2014). Molecular dynamic simulations, using the Na_v_1.4 model, predicted the formation of a hydrogen bond between the Y168-S2 and the R225-S4 residues in the presence of the p.I141V mutation on S1 (Figure 3). Single and double mutants, p.Y168F and p.I141V-Y168F, were generated in order to test these predictions in Na_v_1.5. The functional analyses of these mutants demonstrated the abolition of the functional effects of the p.I141V mutation in the double mutant, consistent with the formation of a specific interaction between Y168-S2 and R225-S4 (Figure 4). The single p.Y168F mutation positively shifted the activation curve, suggesting a compensatory role of these residues on the stability of the voltage-sensing domain.

## Muscular hyper-excitability phenotypes related to an altered activation process of Na_v_1.4—example of myotonia

The skeletal voltage-gated sodium channel Na_v_1.4, encoded by the *SCN4A* gene, is responsible for the initiation of the action potential in muscle fibers, resulting in muscle contraction. Similar to that described for the cardiac sodium channel, the Na_v_1.4 α-subunit is regulated by several proteins, i.e., the β1 subunit that modifies kinetics and gating (Isom, 2001). Similar to the Na_v_1.5 channel, Na_v_1.4 is a large protein composed of four homologous domains (I–IV), each containing six transmembrane helices (S1–S6) (Figure 1). Mutations in the *SCN4A* gene have been associated with altered excitability of skeletal muscle (Jurkat-Rott et al., 2010). The majority of the mutations in Na_v_1.4 were found in the voltage sensor segments S4, the S4-S5 linkers, or in the pore forming segments S5-S6 (Jurkat-Rott et al., 2010). They were found to induce both a loss or gain of function. Gain of function effect has been described to be more frequent (Sokolov et al., 2007; Petitprez et al., 2008; Jurkat-Rott et al., 2010; Corrochano et al., 2014).

Myotonia is one example of skeletal muscle hyper-excitability in which a voluntary contraction or electromechanical stimulation can provoke trains of repetitive action potentials. This causes a delay in relaxation after muscle contraction. This phenotype has been associated with several *SCN4A* mutations that affect the activation and the slow inactivation processes of Na_v_1.4 (Petitprez et al., 2008; Jurkat-Rott et al., 2010; Kokunai et al., 2012; Yoshinaga et al., 2012; Corrochano et al., 2014). Among these *SCN4A* mutants, the substitution of isoleucine to valine in S1-DI and S1-DII affect the biophysical properties of Na_v_1.4 similar to the aforementioned example (Wagner et al., 1997; Petitprez et al., 2008). *In vitro* characterization of the p.I141V and p.I588V mutants demonstrated a negative shift of the voltage dependence of activation in the presence of these mutants (Wagner et al., 1997; Petitprez et al., 2008). *In vivo* characterization of p.I588V knock-in mice demonstrated that these mice suffered from unprovoked intermittent hind-limb immobility attacks. The mice were not able to move their hind-limbs, confirming the implication of this mutation in the occurrence of myotonia (Corrochano et al., 2014).

## Neuronal hyper-excitability phenotypes related to an altered activation process of Na_v_1.7—example of erythromelalgia

The Na_v_1.7 channel is one of the neuronal isoforms of voltage gated sodium channels. It is preferentially expressed in the nociceptive dorsal root ganglia and sympathetic ganglia, and may play an important role in nociception (Sangameswaran et al., 1997; Toledo-Aral et al., 1997; Cummins et al., 1998; Rush et al., 2007). Both gain and loss of function mutations of the *SCN9A* gene, which encodes the Na_v_1.7 α-subunit, have been associated with pain syndromes, including erythromelalgia (Cox et al., 2006; Dib-Hajj et al., 2007).

Inherited erythromelalgia is a rare disorder characterized by recurrent episodes of pain associated with redness and swelling in various parts of the body, particularly the hands and the feet (Drenth and Michiels, 1990). Standing, exercise, or local exposure to heat can induce the symptoms in affected patients. Among the described Na_v_1.7 gain of function mutations that are associated with inherited erythromelalgia (Dib-Hajj et al., 2007; Cheng et al., 2008, 2011; Cregg et al., 2013; Estacion et al., 2013; Vasylyev et al., 2014), the isoleucine to valine substitution (as that described for the cardiac and muscular disorders) was found in a Taiwanese family with the characteristic features of erythromelalgia. Lee *et al* identified the implicated p.I136V mutation in the Na_v_1.7 channel (Lee et al., 2007), which exhibited similar biophysical modifications to the Na_v_1.4-I141V and Na_v_1.5-I141V mutants. The p.I136V mutant shifted the voltage dependence of activation toward more negative potentials, leading to an increase and shift of the sodium window current (Cheng et al., 2008).

## Conclusion

In this review, the comparison between several *Na_v_* mutants that have been linked to cardiac, muscular, and neuronal hyper-excitability phenotypes has revealed: (i) a focused localization of these mutants on the VSD domain, particularly on the S4 arginine residues for cardiac disorders, (ii) an abnormal voltage dependence of activation as a shared biophysical mechanism of the clinical manifestations, and (iii) the functional importance of some highly conserved residues, notably isoleucine 141 for Na_v_1.4 and Na_v_1.5, and the homologous isoleucine 136 in Na_v_1.7.

### Conflict of interest statement

The authors declare that the research was conducted in the absence of any commercial or financial relationships that could be construed as a potential conflict of interest.
